# Восстановление качества эякулята после андрогенной заместительной и комбинированной терапии гипогонадизма

**DOI:** 10.14341/probl13545

**Published:** 2025-09-14

**Authors:** Е. Р. Роживанова, Р. В. Роживанов, Е. Н. Андреева, Г. А. Мельниченко, Н. Г. Мокрышева

**Affiliations:** Национальный медицинский исследовательский центр эндокринологии им академика И.И. Дедова; I.I. Dedov Endocrinology Research Centre; Национальный медицинский исследовательский центр эндокринологии им академика И.И. Дедова; Российский университет медицины; I.I. Dedov Endocrinology Research Centre; Russian University of Medicine

**Keywords:** эякулят, мужчины, гипогонадизм, тестостерон, гонадотропины, ejaculate, men, hypogonadism, testosterone, gonadotropins

## Abstract

**BACKGROUND:**

BACKGROUND: To optimize androgen replacement therapy for male hypogonadism to improve reproductive prospects.

**AIM:**

AIM: To compare the effectiveness of restoring the quality of ejaculate in men receiving androgen replacement therapy (AZT) and patients receiving course combination therapy with testosterone and chorionic gonadotropin (AZT/HG).

**MATERIALS AND METHODS:**

MATERIALS AND METHODS: In observational prospective study was included 53 men observed at The National Medical Research Center for Endocrinology and AZT (n=19) or AZT/HG (n=34) more than 5 years, followed by stimulating gonadotropin therapy. The qualitative parameters of ejaculate were evaluated in all patients. The basic level of statistical significance was p<0,05.

**RESULTS:**

RESULTS: The patient groups were comparable in age, BMI, duration of therapy used, type of testosterone preparation, as well as the etiology of hypogonadism. Sperm concentration in the AZT group there was a statistically significant negative dynamics, while in the ART/HG group, there were no statistically significant differences in the dynamics of sperm concentration. Statistically significant differences in the value of sperm concentration change were revealed. In both groups was observed statistically significant negative dynamics for sperm motility and morphology. There were no statistically significant differences in the value of changes motility and sperm morphology in both studied groups.

**CONCLUSION:**

CONCLUSION: Course combination therapy with testosterone and chorionic gonadotropin is characterized by better results for sperm concentration restoration compared with androgenic replacement therapy. For the restoration of sperm motility and morphology both methods do not show satisfactory results.

## ОБОСНОВАНИЕ

Одна из проблем, связанных с мужским гипогонадизмом, заключается в том, что андрогенная заместительная терапия, являющаяся основным методом его лечения, не улучшает, а часто даже ухудшает репродуктивную функцию мужчин из-за подавления гонадотропной функции гипофиза [1]. Для репродуктивной реабилитации при гипогонадизме применяется комбинированная терапия препаратами гонадотропинов, но она не используется для долгосрочного лечения и показана при лечении бесплодия непосредственно [2]. Однако многие пациенты к моменту развития гипогонадизма еще не имеют репродуктивных планов. В таких ситуациях возможно две тактики лечения: назначение андрогенной заместительной терапии, а далее, при необходимости репродуктивной реабилитации, ее замена на комбинированную терапию гонадотропинами или назначение курсовой комбинированной терапии препаратом тестостерона курсом длительностью 6 месяцев, а далее — хорионического гонадотропина курсом 2 месяца, и при необходимости репродуктивной реабилитации — ее дальнейшая замена на комбинированную терапию гонадотропинами. При этом не установлено, какой из подходов является более предпочтительным в целях восстановления качества эякулята.

## ЦЕЛЬ

Сравнить эффективность восстановления качества эякулята мужчин, получавших андрогенную заместительную терапию (АЗТ), и пациентов, получавших курсовую комбинированную терапию препаратом тестостерона и хорионического гонадотропина (АЗТ/ХГ).

## МАТЕРИАЛЫ И МЕТОДЫ

## Место и время проведения исследования

ГНЦ ФГБУ «НМИЦ Эндокринологии» Министерства здравоохранения России, Москва. Набор пациентов для включения в исследование и их итоговое обследование осуществлялось в период с марта 2006 по ноябрь 2024 гг.

## Изучаемые популяции

В исследование включались пациенты, наблюдающиеся в ГНЦ ФГБУ «НМИЦ Эндокринологии» Министерства здравоохранения России.

Критерии включения: возраст старше 18 лет, мужской пол, андрогенная заместительная (любым препаратом тестостерона) либо курсовая комбинированная терапия любым препаратом тестостерона (курсом 6 мес.) и хорионического гонадотропина (курсом 2 мес.) длительностью более 5 лет, желание репродуктивной реабилитации.

Критерии невключения: первичный (гипергонадотропный) гипогонадизм, любые виды пубертатного и препубертатного гипогонадизма, патологические изменения любого из яичек при осмотре, нарушения кариотипа, задержка полового развития, наличие в анамнезе крипторхизма, вирусного паротита, воспалительные заболевания, опухоли, травмы или хирургических вмешательства на половых органах, лейкоспермия, азооспермия, криптозооспермия, урогенитальные инфекции, варикоцеле, носительство антиспермальных антител, сахарный диабет, гиперпролактинемия, некомпенсированные гипотиреоз, тиреотоксикоз, гиперкортицизм и гипокортицизм, алкоголизм, противопоказания к терапии гонадотропинами (повышенная чувствительность или аллергическая реакция на препарат хорионического гонадотропина или фоллитропина, не устраненные опухоли молочной железы, гипофиза, гипоталамуса, тромбофлебит глубоких вен) [3][4].

Критерии исключения: отказ от выполнения программы исследования (все проводимые исследования являлись рутинными, однако у пациентов было право отказаться от них полностью или частично, и такие пациенты в исследование не включались).

## Способ формирования выборки из изучаемой популяции

Выборка формировалась сплошным способом. Пациенты распределялись по группам, в зависимости от типа получаемой терапии.

## Дизайн исследования

Наблюдательное проспективное нерандомизированное сравнительное исследование.

## Методы

Все включенные в исследование пациенты были обследованы по стандартной единой схеме, включающей сбор жалоб, данных анамнеза, анализ медицинской документации, клиническое обследование. Регистрировались следующие результаты обследования: рост, вес, индекс массы тела (ИМТ), показатели спермограммы. Оценка спермограмм осуществлялась в соответствии с рекомендациями ВОЗ, 2021 г., путем световой микроскопии с помощью микроскопа Olimpus 41 CX (Япония) и камеры Маклера того же производителя [5]. С целью оценки содержания морфологически нормальных форм сперматозоидов проводилось исследование эякулята по точным критериям Крюгера. Исследования проводились двукратно. Первый раз исследование эякулята проводилось при наличии у пациента желания в будущем иметь детей и до назначения АЗТ или АЗТ/ХГ. Если при первичном исследовании эякулята, результаты являлись патологическими, то проводилась терапия, направленная на улучшение его качества в соответствии с патогенезом. В качестве отправной точки в исследовании фиксировался лучший результат спермограммы. Далее назначалась либо АЗТ, либо АЗТ/ХГ. При подборе дозы как препаратов тестостерона, так и хорионического гонадотропина определялся уровень общего тестостерона (референсный интервал (РИ) 12,0–28,2 нмоль/л) методом иммунохемилюминесцентного анализа на автоматическом анализаторе Vitros ECi 3600 (Ortho-Clinical Diagnostics). В качестве завершающей точки в исследовании фиксировался результат спермограммы, полученной после 6 месяцев комбинированной терапии гонадотропинами, назначенной сразу после отмены АЗТ либо АЗТ/ХГ.

## Описание медицинского вмешательства

Осуществлялся забор эякулята в стерильные контейнеры путем мастурбации (половое воздержание 3–5 суток). После отмены АЗТ либо АЗТ/ХГ назначалась комбинированная терапия препаратами хорионического гонадотропина в индивидуально подобранной дозе, которая составила от 1000 до 3000 ЕД 1 раз в 3 дня внутримышечно и препарат рекомбинантного фолликулостимулирующего гормона в дозе 75 ЕД подкожно через день. Доза хорионического гонадотропина определялась путем оценки уровня общего тестостерона утром натощак, на следующий день после инъекции препарата (целевым значением являлось 15–25 нмоль/л). Длительность вмешательства составила 6 месяцев.

## Статистический анализ

Принципы расчета размера выборки

Размер выборки предварительно не рассчитывался.

Методы статистического анализа данных

Статистическая обработка полученных данных была проведена с использованием пакета прикладных программ STATISTICA (Stat Soft Inc., США, версия 10.0). Сравнение по качественным признакам осуществлялось непараметрическим методом χ² с поправкой Йетса, по количественным — U тестом Манна-Уитни для независимых групп и тестом Вилкоксона для зависимых. Базовый пороговый уровень значимости p<0,05. При множественных сравнениях применялась поправка Бонферрони. Результаты исследований представлены в виде медиан параметров, интерквартильного отрезка для количественных признаков, а также абсолютных чисел и процентов для качественных признаков.

## Этическая экспертиза

Проведение исследования одобрено локальным этическим комитетом ГНЦ ФГБУ «НМИЦ Эндокринологии» Министерства здравоохранения России (протокол №16 от 11.09.2024).

## РЕЗУЛЬТАТЫ

Характеристики выборки пациентов представлены в таблице 1.

**Table:** Таблица 1. Характеристики выборки Примечание: АЗТ — андрогенная заместительная терапия; АЗТ/ХГ — курсовая комбинированная терапия препаратом тестостерона и хорионическим гонадотропином; ИМТ — индекс массы тела. Me [ 25%; 75%], U тест Манна-Уитни — для количественных признаков, χ² с поправкой Йетса — для качественных. Уровень значимости p<0,05.

Показатель	АЗТ (n=19)	АЗТ/ХГ (n=34)	p
Возраст, лет	38 [ 36; 41]	39 [ 36; 44]	0,492
ИМТ, кг/м²	27,1 [ 26,2; 28,2]	26,6 [ 25,9; 27,3]	0,203
Длительность терапии, лет	6 [ 6; 7]	6 [ 6; 8]	0,522
Применяемый препарат тестостерона	Трансдермальный гель — 31,6% Пролонгированный инъекционный — 68,4%	Трансдермальный гель — 32,3% Пролонгированный инъекционный — 67,6%	0,803
Этиология гипогонадизма	Гипогонадотропный гипогонадизм вследствие аденомы гипофиза — 26,3% Гипогонадотропный гипогонадизм вследствие краниофарингиомы — 15,8% Нормогонадотропный гипогонадизм ассоциированный с метаболическими нарушениями — 36,8% Возрастной нормогонадотропный гипогонадизм — 21,0%	Гипогонадотропный гипогонадизм вследствие аденомы гипофиза — 32,3% Гипогонадотропный гипогонадизм вследствие краниофарингиомы — 8,8% Нормогонадотропный гипогонадизм ассоциированный с метаболическими нарушениями — 32,3% Возрастной нормогонадотропный гипогонадизм — 26,5%	-

Группы пациентов были сопоставимы по возрасту, ИМТ, длительности применяемой терапии, типу препарата тестостерона, этиологии гипогонадизма, а также исходным показателям качества эякулята.

Результаты обследования пациентов в динамике представлены в таблице 2.

**Table:** Таблица 2. Результаты обследования пациентов Примечание: АЗТ — андрогенная заместительная терапия; АЗТ/ХГ — курсовая комбинированная терапия препаратом тестостерона и хорионическим гонадотропином. Me [ 25%; 75%], *U тест Манна-Уитни, проводились множественные сравнения, применялась поправка Бонферрони, уровень статистической значимости p<0,0055. **тест Вилкоксона, проводились множественные сравнения, применялась поправка Бонферрони, уровень статистической значимости p<0,0166.

Показатель	АЗТ (n=19)	АЗТ/ХГ (n=34)	p*
Кол-во (концентрация) сперматозоидов в 1 мл, млн исходно	72 [ 31; 111]	74 [ 39; 92]	0,562
Кол-во (концентрация) сперматозоидов в 1 мл, млн динамика	67 [ 22 ;94]	75 [ 49; 96]	0,600
p**	0,0101	0,043	
Изменение величины исследуемого параметра	-4 [ -9; 2]	1 [ -2; 10]	0,0035
Подвижность сперматозоидов (А + В), % исходно	33 [ 28; 47]	38 [ 31; 45]	0,992
Подвижность сперматозоидов (А + В), % динамика	18 [ 16; 29]	22 [ 16; 36]	0,294
p**	0,0015	0,0000	
Изменение величины исследуемого параметра	-16 [ -29; -4]	-9 [ -20; -3]	0,245
Нормальные формы сперматозоидов, % исходно	10 [ 3; 12]	5 [ 4; 8]	0,599
Нормальные формы сперматозоидов, % динамика	4 [ 2; 9]	3 [ 2; 5]	0,246
p**	0,0026	0,0000	
Изменение величины исследуемого параметра	-1 [ -4; 0]	-2 [ -3; -1]	0,490

В отношении концентрации сперматозоидов в группе АЗТ отмечалась статистически значимая отрицательная динамика, в то время как в группе АЗТ/ХГ статистически значимых различий в динамике концентрации сперматозоидов выявлено не было. При этом были выявлены статистически значимые различия в величине изменения концентрации сперматозоидов в исследуемых группах. В отношении подвижности и морфологии сперматозоидов в обеих группах отмечалась статистически значимая отрицательная динамика. Статистически значимых различий в величинах изменений как показателей подвижности, так и морфологии сперматозоидов в исследуемых группах выявлено не было.

## ОБСУЖДЕНИЕ

## Репрезентативность выборок

Учитывая, что выборка пациентов была мала и сформирована исключительно в ГНЦ ФГБУ «НМИЦ Эндокринологии» Министерства здравоохранения России, характеристики в других выборках могут отличаться.

## Сопоставление с другими публикациями

Согласно результатам, полученным в исследовании, установлено, что после АЗТ/ХГ концентрация сперматозоидов восстанавливается лучше, чем после АЗТ. Наличие данного эффекта может быть обусловлено тем, что периодическое курсовое применение хорионического гонадотропина препятствует атрофии тестикулярной ткани [6][7], которая может наблюдаться при монотерапии препаратами тестостерона [8]. Другими исследователями также было установлено, что применение хорионического гонадотропина положительно сказывается на увеличении выработки сперматозоидов, даже без применения препаратов фолликулостимулирующего гормона [9][10]. Оптимальной терапией для улучшения качества эякулята при мужском гипогонадизме является сочетание препаратов хорионического гонадотропина и фолликулостимулирующего гормона [11–13]. Однако эта терапия не рекомендуется в качестве долгосрочной или постоянной, и рекомендуемая ее длительность составляет от 3 до 12 месяцев [12][13]. Но для повышения ее эффективности в будущей репродуктивной реабилитации, согласно результатам нашего исследования, включение в схему АЗТ препарата хорионического гонадотропина короткими курсами является целесообразным. В отношении восстановления подвижности или морфологии сперматозоидов нами не было отмечено преимуществ одного вида терапии над другим, что объяснимо тем, что применение хорионического гонадотропина самого по себе не улучшает морфологию или подвижность сперматозоидов [8][13][14]. Так, в метаанализе Rastrelli G. и соавт. (2014 г.) применение хорионического гонадотропина увеличивало лишь концентрацию сперматозоидов, но никак не сказывалось на числе подвижных и морфологически нормальных форм [14]. Ухудшение подвижности и морфологии сперматозоидов в динамике вне зависимости от схемы лечения, наблюдаемое в нашем исследовании, можно объяснить возрастными факторами. Так как длительность терапии составила в целом по выборке от 5 до 9 лет, соответственно, пациенты и постарели на тот же период, что сказалось на сперматогенезе. Негативное влияние возраста на качество эякулята подтверждено результатами работ многих авторов [15–17]. Большинством исследователей выделяются следующие возрастные факторы ухудшения сперматогенеза — окислительный стресс, истощение сперматогониальных стволовых клеток, мутации de novo в половых клетках, возникающих в результате множественных митотических делений, повреждение и нарушения процессов метилирования генетического материала, а также увеличение длины теломер в сперматозоидах [15–19]. Эти факторы могли оказать негативную роль и на исследуемую нами популяцию, что обусловило ухудшение подвижности и морфологии сперматозоидов в динамике.

## Клиническая значимость результатов

Установленные лучшие результаты курсовой комбинированной терапии препаратом тестостерона и хорионического гонадотропина в отношении последующего восстановления концентрации сперматозоидов свидетельствуют о предпочтительности этого метода при долгосрочном лечении гипогонадизма в целях дальнейшей репродуктивной реабилитации. Так как отмечается отрицательная динамика в отношении подвижности и морфологии сперматозоидов вне зависимости от выбранного метода, а также отрицательная динамика концентрации сперматозоидов при использовании АЗТ, целесообразно мотивировать мужчин с гипогонадизмом на максимально раннее проведение репродуктивной реабилитации.

## Ограничения исследования

Учитывая, что выборка пациентов была сформирована исключительно в ГНЦ ФГБУ НМИЦ эндокринологии Минздрава РФ, характеристики других выборок могут отличаться. Поскольку рандомизации не проводилось, в исследованиях с большей степенью доказательности выводы могут измениться.

## Направления дальнейших исследований

Проведение рандомизированного проспективного сравнительного исследования достаточной мощности.

## ЗАКЛЮЧЕНИЕ

Курсовая комбинированная терапия препаратом тестостерона и хорионического гонадотропина характеризуется лучшими результатами в отношении последующего восстановления концентрации сперматозоидов по сравнению с андрогенной заместительной терапией. В отношении восстановления подвижности и морфологии сперматозоидов оба метода не демонстрируют удовлетворительных результатов.

## ДОПОЛНИТЕЛЬНАЯ ИНФОРМАЦИЯ

Источник финансирования. Работа выполнена по инициативе авторов без привлечения финансирования.

Конфликт интересов. Авторы данной статьи подтвердили отсутствие конфликта интересов, о котором необходимо сообщить.

Участие авторов. Все авторы одобрили финальную версию статьи перед публикацией, выразили согласие нести ответственность за все аспекты работы, подразумевающую надлежащее изучение и решение вопросов, связанных с точностью или добросовестностью любой части работы.

Благодарности. Авторы выражают искреннюю благодарность пациентам, принявшим участие в проведении исследования.
